# Occurrence and seasonal variability of Dense Shelf Water Cascades along Australian continental shelves

**DOI:** 10.1038/s41598-020-66711-5

**Published:** 2020-06-16

**Authors:** Tanziha Mahjabin, Charitha Pattiaratchi, Yasha Hetzel

**Affiliations:** Oceans Graduate School and The UWA Oceans Institute, The University of Western Australia, Perth, WA 6009 Australia

**Keywords:** Physical oceanography, Physical oceanography

## Abstract

Transport of water between the coast and the deeper ocean, across the continental shelf, is an important process for the distribution of biota, nutrients, suspended and dissolved material on the shelf. Presence of denser water on the inner continental shelf results in a cross-shelf density gradient that drives a gravitational circulation with offshore transport of denser water along the sea bed that is defined as Dense Shelf Water Cascade (DSWC). Analysis of field data, collected from multiple ocean glider data missions around Australia, confirmed that under a range of wind and tidal conditions, DSWC was a regular occurrence during autumn and winter months over a coastline spanning > 10,000 km. It is shown that even in the presence of relatively high wind- and tidal-induced vertical mixing, DSWCs were present due to the strength of the cross-shelf density gradient. The occurrence of DSWC around Australia is unique with continental scale forcing through air-sea fluxes that overcome local wind and tidal forcing. It is shown that DSWC acts as a conduit to transport suspended material across the continental shelf and is a critical process that influences water quality on the inner continental shelf.

## Introduction

Globally, the coastal ocean is the receiving basin for input of suspended and dissolved matter that includes nutrients, biota and pollutants and represents an important component of the ocean environment, connecting the terrestrial system to the deeper ocean^1^. Transport processes that contribute to the exchange of water between the coast and deeper regions: cross-shelf exchange, has been defined as one of the central problems in coastal physical oceanography^2^. Majority of inputs to the coastal zone are through fresh water sources such as rivers and anthropogenic discharges. As these inputs are usually buoyant, they are transported along the ocean surface layer subject to the prevailing hydrodynamic regime and are easily identified in the field and in satellite imagery. If the spatial scale is unrestricted by coastal features and under relatively low mixing conditions, river plumes can influence the hydrodynamics to hundreds of kilometres from the source region^3,4^. These systems have been subject to many investigations over the past decades being defined as river plumes or Regions of Freshwater Influence (ROFI) where the hydrodynamic regime is governed by the positive density gradient between lower salinity riverine water and higher salinity oceanic water^3^. In ROFI, buoyancy input from freshwater sources exceeds that from changes in heat and freshwater fluxes across the ocean surface.

**Figure 1:**
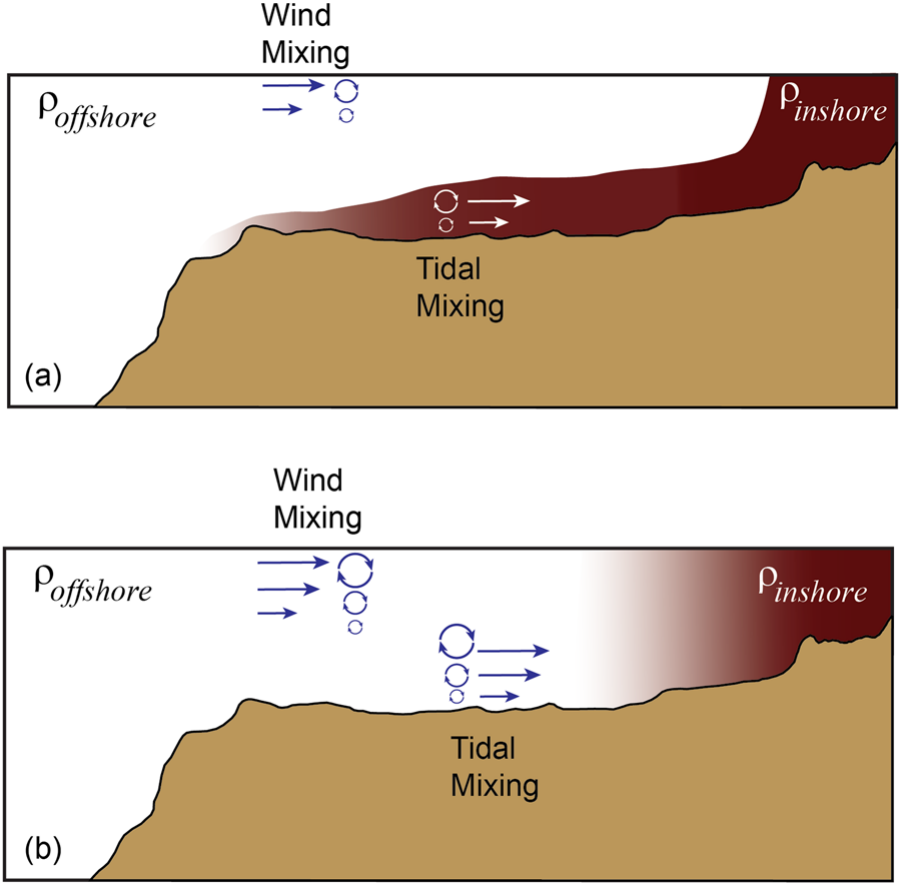
Schematic of the influence of wind- and tide- induced vertical mixing on the continental shelf in the presence of a cross-shelf density gradient: (**a**) Under low wind- and tidal- induced vertical mixing, a dense shelf water cascade is present; (**b**) Under strong vertical mixing, the water column is well mixed although a density gradient is present (modified from Mahjabin *et al*.^8^).

Regions that experience a Mediterranean climate (dry hot summers, mild cooler winters and low precipitation) are subject to high rates of evaporation and negligible river input resulting in a net loss of fresh water from the coastal region. Therefore, coastal water bodies become more saline than the surrounding ocean water with density decreasing from the coast to the ocean (i.e. a negative density gradient). With the advent of cooling during late autumn and winter, this density contrast is increased. Here, buoyancy input from heat and freshwater fluxes across the ocean exceed that due to freshwater sources and thus are opposite to that in ROFI regions where the density is increasing from the coast to the ocean. Rather than having a buoyant plume due to freshwater input, the negative density gradient drives an offshore-directed flow of denser water along the seabed (Fig. 1)^5–8^. A similar feature to that observed in ‘inverse’ estuarine systems located in Mediterranean climates with negligible or intermittent freshwater input and loss of freshwater through evaporation^9,10^. In coastal regions, the buoyancy-driven gravity current is defined as Dense Shelf Water Cascade (DSWC)^5,8,11–14^ and has mainly been considered as a high‐latitude process^11,13^. DSWCs documented around the Mediterranean Sea are episodic and their formation related to specific atmospheric conditions. For example, in the northern Adriatic Sea, DSWC occurs only in years where there is substantial cooling of the whole water column associated with wintertime bora winds, preconditioned by a lower-than-usual river discharges^15^.

Evidence for DSWC has been provided in locations around Australia based on single field experiments lasting over a period of about 1 month^6–9,13,14,16–18^. Majority of these studies examined the export of higher salinity water from large inverse estuary systems^6,7,16,18^ with only two studies examining shelf regions^13,17^. Seasonal variation in DSWC was identified as a major feature through the deployment of ocean gliders along the Rottnest continental shelf^5^. This paper is an extension of the work by Pattiaratchi *et al*.^5^ from a single location and season to multiple locations and seasons to examine the generating mechanisms and seasonal variability of DSWC formation around Australia spanning a coastline > 10,000 km and a range of wind and tidal conditions.

**Figure 2:**
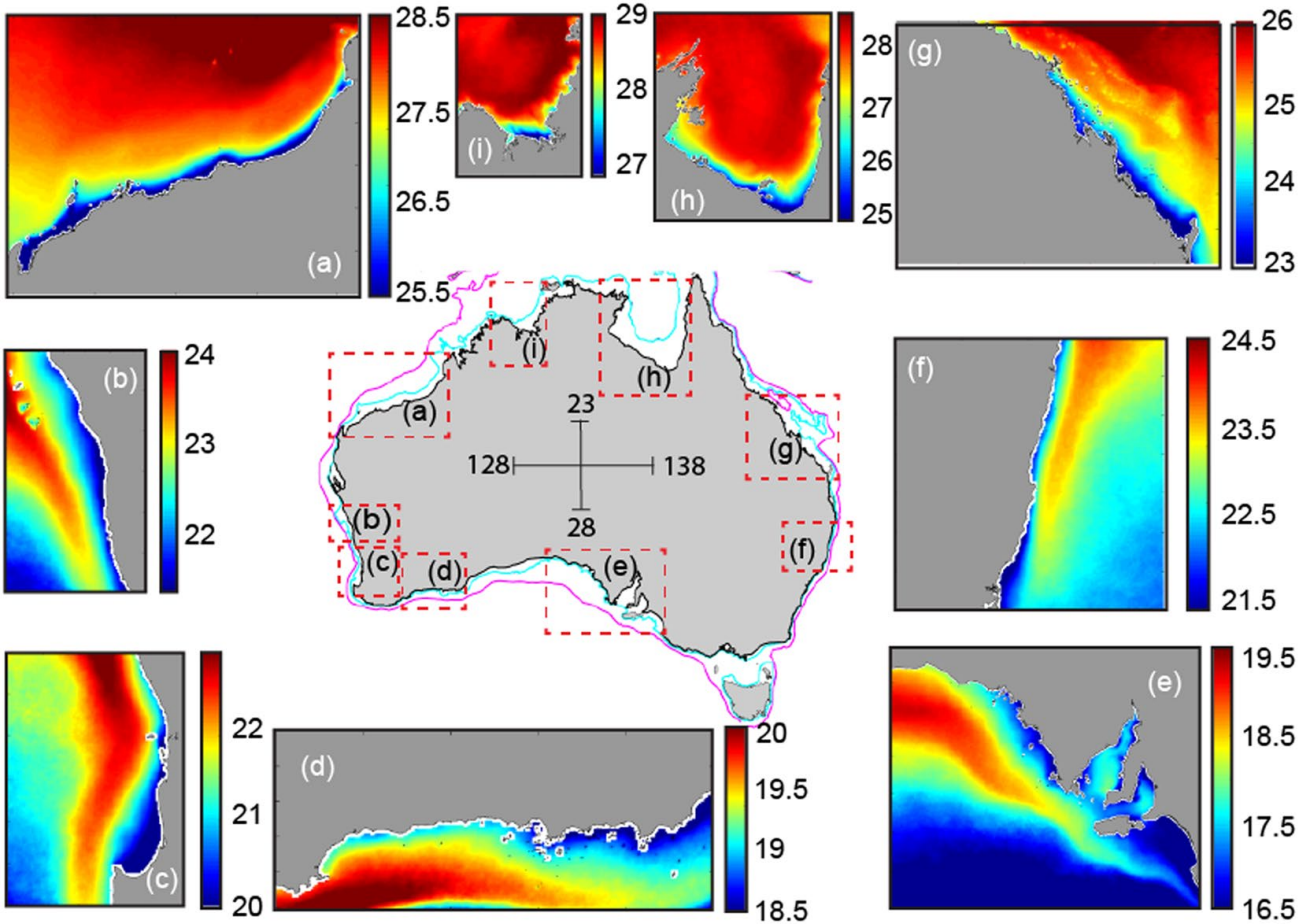
Sea Surface Temperature (SST) climatology during May and June around Australian continental shelves showing a band of cooler water at the coast. The climatology is described in Wijffels *et al*.^20^ included data collected over a 25 years period. Note the different temperature scales.

The oceans surrounding Australia experience some of the highest evaporation (>2.5 m per annum^19^) and negative heat flux (cooling) rates globally. Climatological means of the freshwater budget (evaporation-precipitation) and the net heat flux indicate that from January to June the mean evaporation rates are ~ 6 mmday^−1^ (Figure S1) with the spatial extent of the high evaporation regions during May-June (late autumn). In contrast, the net heat flux is positive (~ 750 Wm^−2^) in January-February and changes to a negative heat flux (<−1000 Wm^−2^) in May-June. High rates of cooling are highlighted by the sea surface temperature climatology in Australian shelf waters^20^ during May and June that indicate a band of colder water closer to the coast (Fig. 2). Due to high evaporation rates (Figure S1), these coastal waters also contain elevated salinities. Combination of higher salinity and colder water at the coast lead to higher density water on the inner continental shelf creating horizontal density gradients that drive DSWC. In this paper, occurrence of DSWC around Australia and their seasonal variability, both spatially and temporally, are examined using an extensive ocean glider data set obtained between 2008 and 2019^21^ that included 254 cross-shelf transects selected from over 126 glider missions from eight different regions. The main aims of this paper are to (1) identify and document the occurrence of DSWC around Australia; and, (2) define the main controlling mechanisms and their seasonal variability. The extensive data set, collected using ocean gliders, included large spatial scales (>10,000 km) and enabled the identification of seasonal (monthly) variability. DSWC were observed at different locations independent of the wind and tidal regime due to the strength of the negative cross-shelf density gradient (denser water along the coast).

### Theory

Circulation and mixing on continental shelves are largely driven by wind, tides and input of buoyancy at the boundaries (terrestrial and the air-sea interface). In the shallow continental shelves considered here, with mean water depths less than the Ekman depth (at Two Rocks, the Ekman depth is 70 m whilst mean water depth is 40 m) the influence of earth rotation is minimal^1^. Circulation patterns resulting from interactions between these mechanisms together with complex topography of continental shelves are challenging to measure and to unravel. Numerical simulations that include idealised setups have been used to isolate the role of different processes and to develop predictive capability^22,23^. However, simple theories for important aspects of buoyancy-influenced coastal currents have been developed and evaluated with field campaigns and numerical models that have contributed to a broad understanding of coastal dynamics.

Vertical stratification in the coastal ocean is controlled by buoyancy fluxes that promotes stratification whilst turbulent kinetic energy generated through wind and tides promotes mixing^24,25^. The competition between buoyancy and mixing inputs determine stratification status of the water column. Buoyancy fluxes include horizontal advection of water, due to a longitudinal density gradient $(\frac{\partial \rho }{\partial x})$, from a region of higher buoyancy over denser water (e.g. river plumes) or lower buoyancy along the bottom (e.g. DSWC) by a vertically sheared flow. This mechanism was termed “straining” by Simpson *et al*.^24^. Ratio of the longitudinal density gradient (stratifying force) to vertical mixing (destratifying force) is defined as the horizontal Richardson number (Ri_x_), also defined as the Simpson number^24,26,27^:

1 $$R{i}_{x}\approx \frac{g}{\rho }\frac{{h}^{2}}{{u}_{\ast }^{2}}\frac{\partial \rho }{\partial x}$$

where, h = mean water depth; ρ = mean seawater density; and, u_*_ = friction velocity; Monismith *et al*.^26^ proposed that values of *Ri*_*x*_ > O(1) reflected a dominance of stratification by horizontal density gradients over tidal mixing, resulting in a stratified water column. Burchard^28^ modified Eq. (1) to include wind mixing:

2 $$R{i}_{x}\approx \frac{g}{\rho }\frac{{h}^{2}}{{u}_{\ast wt}^{2}}\frac{\partial \rho }{\partial x}$$

with ${u}_{\ast wt}^{2}={u}_{\ast t}^{2}+{u}_{\ast w}^{2}$ and ${u}_{\ast t}^{2}={k}_{D}{u}_{b}^{2}$ and ${u}_{\ast w}^{2}={k}_{s}\frac{{\rho }_{a}}{\rho }{W}^{2}$. Here, *k*_*D*_ and *k*_*s*_ are the bottom and surface drag coefficients, respectively and *ρ*_*a*_ is the density of air and U is the near bed velocity (tidal) and W is the wind speed.

Numerical simulations by Horwitz^29^ indicated that cross-shelf transport was controlled by the horizontal Richardson number (*Ri*_*x*_) and found that for increasing density in the direction of the wind stress (for this study onshore winds), the horizontal density gradient increased the cross-shelf transport by strengthening the stratification and reducing mixing. For wind stress opposing the density gradient, the cross-shelf density gradient decreased the cross-shelf transport by enhancing vertical mixing.

DSWCs are generated through the formation of a cross-shelf density gradient by surface cooling and/or evaporation^5^. Consider an initially homogeneous water body on a continental shelf with a sloping bathymetry subject to uniform surface cooling (or heating). Here, the shallower water cools (heats) more rapidly compared to those in deeper water (‘differential heating/cooling’) that results in the formation of a cross-shelf density gradient^30^. The density gradient provides the buoyancy input that drives the DSWC. In contrast, turbulent kinetic energy resulting from wind and/or tidal action promotes vertical mixing. When the buoyancy input dominates over turbulent wind and/or tidal mixing, a DSWC is formed (Fig. 1a). In the case of vertical mixing being stronger than the buoyancy input, a vertically mixed water column is present although the cross-shelf density gradient still exists (Fig. 1b).

In the relatively narrow shallow continental shelf regions considered in this study, the dynamical balance consists of buoyancy, Coriolis, wind and tidal induced vertical mixing and friction. This results in the DSWC being moved offshore and deflected to the left at some angle to the coastline^23^. Majority of the locations contained relatively uniform bathymetry with a slight offshore gradient. There were no major topographic features (e.g. reefs, islands etc) in regions where glider deployments were made mainly due to the requirement for safe operation of the gliders.

The potential energy anomaly (*ϕ*) method proposed by Simpson *et al*.^24^ was used here to investigate physical processes governing the DSWC. *ϕ* values express the vertical stability of the water column and is the amount of energy per unit volume (J m^−3^) required to vertically mix a stratified water column:

3 $$\phi =\frac{g}{h}{\int }_{-h}^{0}(\bar{\rho }-\rho )zdz$$

with:

4 $$\bar{\rho }=\frac{1}{h}{\int }_{-h}^{0}\rho (z)dz$$

where, g is the gravitational acceleration (ms^−2^), z is the vertical coordinate pointing upward from the seabed, $\bar{\rho }$ is the depth-averaged density (kgm^−3^) and ρ(z) is the seawater density (kgm^−3^) at depth z, and h is the total water depth (m).

Whilst *ϕ* explains the instant state of water column stability, the temporal change in *ϕ* reflects different processes that are related to advection, vertical mixing through wind and tidal stirring and stratifying mechanisms through buoyancy input^24^.

Burchard and Hofmeister^31^ derived a time-dependent equation for *ϕ* based on the advection of potential temperature and salinity equations, the continuity equation and an equation of state for the potential density. The derived equations, that are more suitable for the analysis of three-dimensional numerical model output represented changes in *ϕ* through advection by currents, vertical mixing, buoyancy fluxes, sinks and sources of potential density and horizontal eddy diffusive density fluxes (for further explanation of these terms and the detailed derivation see Burchard and Hofmeister^31^, de Boer *et al*.^32^ and Kokkos and Sylaios^33^).

We follow the approach of Simpson *et al*.^24^ and describe the temporal change of *ϕ* due to mechanical mixing (wind and tidal stirring) and stratifying mechanisms (e.g. air-sea heat flux and buoyancy flux):

5 $${\left[\frac{\partial \phi }{\partial {\rm{t}}}\right]}_{total}={\left[\frac{4{{\rm{\varepsilon }}{\rm{k}}}_{{\rm{D}}}{\rm{\rho }}}{3{\rm{\pi }}}\frac{|{{{\rm{u}}}_{{\rm{b}}}}^{3}|}{{\rm{h}}}\right]}_{tidal}+{\left[{{\rm{\delta }}{\rm{k}}}_{{\rm{S}}}{{\rm{\rho }}}_{{\rm{a}}}\frac{|{{\rm{W}}}^{3}|}{{\rm{h}}}\right]}_{wind}+{\left[\frac{1}{320}\frac{{{\rm{g}}}^{2}{{\rm{h}}}^{4}}{{{\rm{\rho }}{\rm{N}}}_{{\rm{z}}}}{\frac{\partial {\rm{\rho }}}{\partial {\rm{x}}}}^{2}\right]}_{gravitational}$$

where, the first and second terms on the right represents the mechanical mixing due to the action of tides and wind, respectively. The third term is the advection of buoyancy flux represented by a horizontal density gradient defined as the gravitational circulation^24^. Values for different coefficients are given by Simpson *et al*.^24^ and Nahas *et al*.^16^. δ and ε represent the efficiency of conversion of wind and the tidally-generated turbulent kinetic energy into potential energy (δ = 0.039; ε = 0.0038); k_D_ and k_s_ are the surface and bottom drag coefficients (k_D_ = 2.5 × 10^−3^; k_s_ = 6.4 × 10^−5^); ρ_a_ is the density of air (1.2 kgm^−3^); u_b_ and W represent bottom current and wind speeds (ms^−1^), respectively. The third term defines the buoyancy flux through the advection of higher density water due to the cross-shelf density gradient. N_Z_ is the vertical eddy viscosity coefficient assumed to be 1~ 17 × 10^–5^ m^2^s^−1^ (see also Mahjabin *et al*. ^30^).

When the change in potential energy anomaly (*ϕ*) over time is positive (*dϕ*/*dt* > 0), the water column is stable and stratified, and when *dϕ*/*dt* < 0 the water column is vertically mixed and when *ϕ* = 0, it is vertically homogeneous with the input of buoyancy exactly balanced by vertical mixing. For the water column to be vertically stratified the necessary condition is:

6 $${\left[\frac{1}{320}\frac{{{\rm{g}}}^{2}{{\rm{h}}}^{4}}{{{\rm{\rho }}{\rm{N}}}_{{\rm{z}}}}{\frac{\partial {\rm{\rho }}}{\partial {\rm{x}}}}^{2}\right]}_{gravitational} > {\left[\frac{4{{\rm{\varepsilon }}{\rm{k}}}_{{\rm{D}}}{\rm{\rho }}}{3{\rm{\pi }}}\frac{{{{\rm{u}}}_{{\rm{b}}}}^{3}}{{\rm{h}}}\right]}_{tidal}+{\left[{{\rm{\delta }}{\rm{k}}}_{{\rm{S}}}{{\rm{\rho }}}_{{\rm{a}}}\frac{{{\rm{W}}}^{3}}{{\rm{h}}}\right]}_{wind}$$

Equation (6) implies that when the horizontal density gradient $(\frac{\partial \rho }{\partial x})$is able to overcome vertical mixing through tidal and wind action, DWSC should occur. In contrast if the action of tidal- and/or wind- induced vertical mixing was higher (i.e., higher values of U and/or W) the water column will be vertically mixed thus inhibiting the formation of DSWC.

## Results

**Figure 3:**
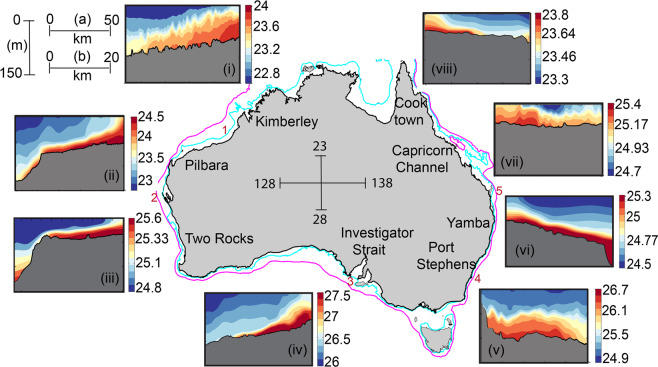
The occurrence of DSWC at the eight different locations around Australia: (i) Kimberley (Aug 2014); (ii) Pilbara (July 2012); (iii) Two Rocks (May 2016); (iv) Investigator Strait (June 2009); (v) Port Stephens (Nov 2008); (vi) Yamba (June 2015); (vii) Capricorn Channel (July 2011); and (viii) Cooktown (July 2016). Vertical and horizontal scales are given at the top left corner. All sigma-t (σ_T_) plots have different colour scales but the same vertical depth scale (maximum 150 m). Kimberley, Pilbara, Investigator Strait, Port Stephens and Capricorn Channel are to 50 km from the shore (horizontal scale (**a**)); and, Two Rocks, Yamba and Cooktown are to 20 km (horizontal scale (**b**)). Note that these are the snapshots of a representative single transect from each location. Previous study locations are indicated in the map: (1) North-West shelf^8,13,14^; (2) Shark Bay^6,7,16^; (3) Spencer Gulf^52,53^; (4) Jervis Bay^17^; (5) Hervey Bay^18^. The DSWC is characterized by the wedge-shaped higher density water with larger thickness close to the coast.

Ocean glider data collected between 2008 and 2019 from 8 different locations around the Australian continent spanning a coastline > 10,000 km (Fig. 3) were analysed and included 254 transects selected from 126 separate ocean glider deployments. The results are presented to highlight the DSWC events as measured from the Slocum ocean glider observations along cross-shelf transects and include: typical examples of DSWC from Two Rocks and the Pilbara; occurrence of DSWC around Australia, seasonality of DSWC; and, examination of the driving mechanisms. Although all the data were used to calculate the cross-shelf density gradients, only selected profiles are presented here.

**Figure 4:**
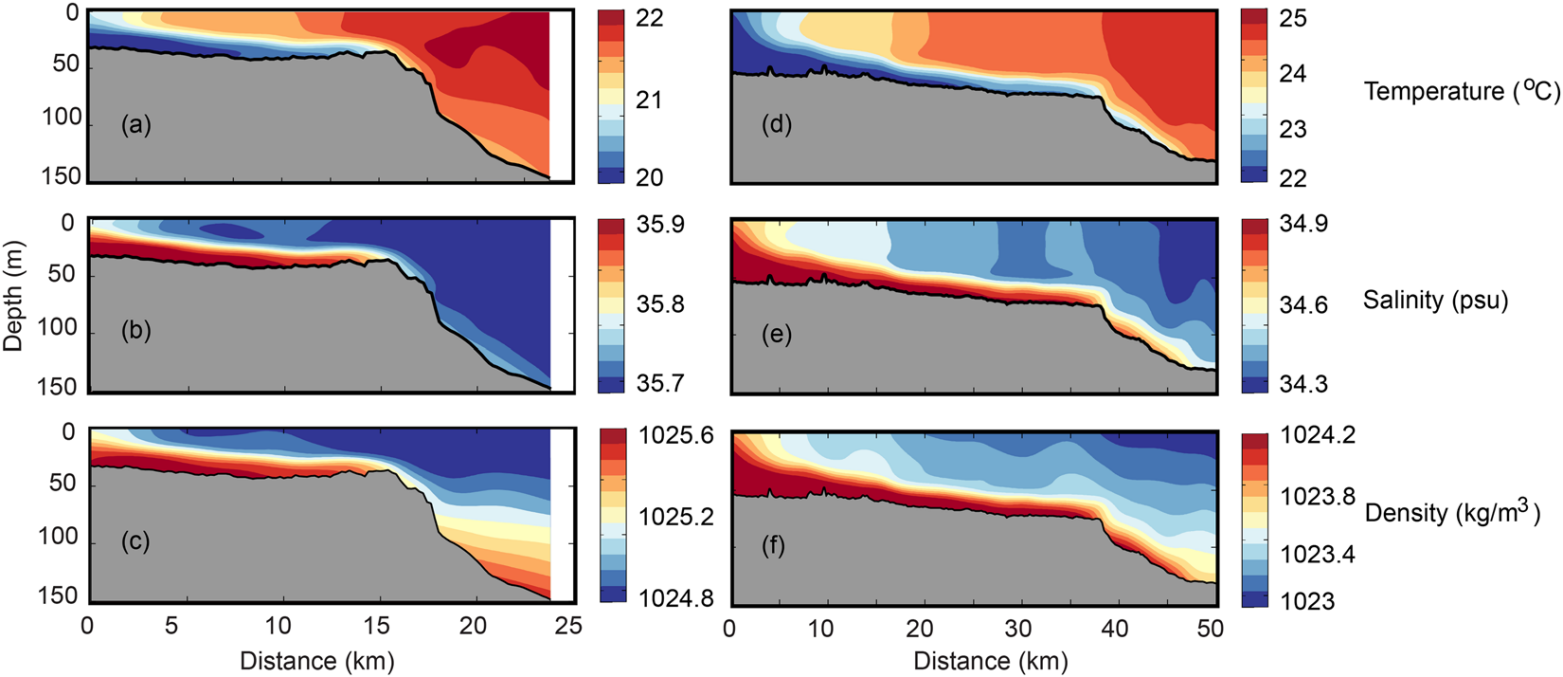
Cross-shelf profiles of temperature, salinity and density at: (**a-c**) Two Rocks, May 2016; and, (**d-f**) Pilbara, July 2012. Note different horizontal scale for each parameter.

Typical cross-shelf transects for Two Rocks (May 2016) and Pilbara (July 2012) indicated that both temperature and salinity contributed to the dense water formation (Fig. 4). At Two Rocks, the difference between inshore and offshore waters for temperature and salinity was 2 °C and 0.2 units, respectively whilst in the Pilbara the difference was 2.5 °C and 0.6 units. At both locations, the DSWC occupied the bottom 20–30 m of the water column and extended to water depths 80 m and 150 m at Two Rocks and Pilbara, respectively. Here, the denser water along the seabed contained cooler, more saline water originating from the shallower inner shelf region. In the relatively narrow and shallow shelves considered here and in the absence of any major river inputs there were no multiple water masses with similar density.

The SST climatology for May/June indicated a narrow band of colder water adjacent to the coast due to heat loss in the shallow waters (Fig. 2). Ocean glider data, collected during the same period, indicated the continuation of this cooler water along the seabed extending further offshore. Higher salinity water is derived from evaporation during summer/autumn (Figure S1). Thus, during this period both temperature (cooling) and salinity (evaporation) contributed to the formation of the DSWC.

Evidence of DSWCs were found in all ocean glider deployments conducted north of 36°S around the Australian coastline, particularly during the winter months. At all of these locations, temperature was the main driver (as seen by the cooler water at the coast, Fig. 2). The locations consisted of a range of tidal and wind forcing: tidal ranges ranged from 0.6 m at Two Rocks to 10 m in the Kimberley whilst the maximum wind speeds ranged from 5.5 ms^−1^ to > 15 ms^−1^ at Two Rocks and Investigator Strait (Table 1). Also at all these locations, freshwater discharge was minimal during the austral late autumn and winter months. The DSWCs extend to a distance > 50 km (Kimberley, Fig. 3i) and to depths > 150 m (Pilbara and Yamba, Figures 3ii,vi). The height of the DSWC was a maximum (>50 m) in the Kimberley whilst at all of the other locations the thickness varied between 20–30 m (Fig. 3).

**Table 1:** Tidal and wind speed range and the horizontal density gradient calculated from glider data for each study site.

Study area	Maximum Current Speed (m/s) Tidal range (m)	Maximum Wind speed (Mean Wind Speed) (m/s)	Horizontal density gradient range (x10^–5^ kgm^−4^)	Simpson Number (Ri_x_) range
(1) Kimberley	0.57 (7–10)	10.6 (5.1)	−0.927 to +0.487	0.01–0.88
(2) Pilbara	0.21 (4–5)	9 (5.9)	−1.423 to +0.829	1.30–0.574
(3) Two Rocks	0.02 (0.6–0.8)	15 (6)	−3.0 to +0.973	0.01–59.09
(4) Investigator Strait	0.13 (1.5–2)	16.5 (6.9)	−1.878 to +2.258	0.05–7.31
(5) Port Stephens	0.03 (2.2–2.5)	6.8 (6.3)	−0.366	3.01–3.41
(6) Yamba	0.26 (1.2)	12.4 (7.7)	−0.44	0.04–1.51
(7) Capricorn Channel	0.32 (6–7)	5.4 (5.4)	−0.982	0.18–1.09
(8) Cook Town	0.24 (2.5–7)	9.3 (7)	−0.817 to +1.873	0.51

### Seasonal variability

**Figure 5:**
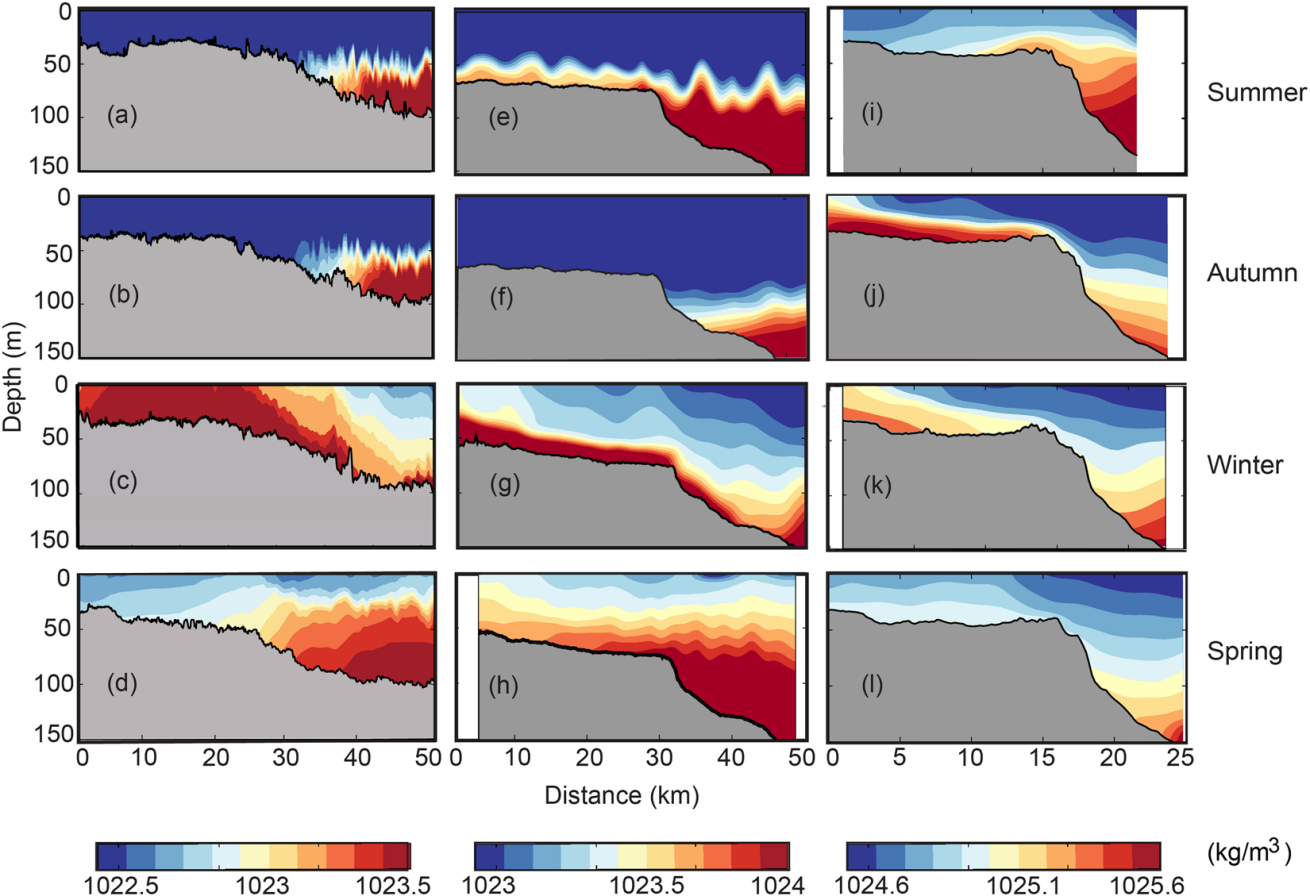
Seasonal cross-shelf density profiles: (**a-d**) Kimberley (Feb 2014; Mar 2015; Aug 2014; and Sep 2012 respectively); (**e-h**) Pilbara (Feb 2014; Mar 2013; July 2012; and Sep 2013 respectively); and, (**i-l**) Two Rocks (Feb 2015; May 2016; Aug 2014; Sep 2011 respectively). All transects at each location contain the same density scale. The horizontal scale at Two Rocks (to 25 km) is different to that Pilbara and Kimberley (to 50 km).

Majority of the ocean glider data were collected from Kimberley, Pilbara, Two Rocks, Investigator Strait and the Great Barrier Reef (Fig. 5) and seasonal cross-shelf density transects from the first 3 locations are presented in Fig. 4. Seasonal variability in these three locations was due mainly to changes in the wind field and air-sea fluxes. The winds are stronger during the summer months due to synoptic winds and strong sea breezes. Similarly, there was strong solar heating/evaporation during the summer months and cooling of the shallower water during late autumn/winter months (Fig. 2 and S1, see Supplementary). This variability was reflected in the seasonal cross-shelf transects. During the summer months, stronger winds mixed the upper ocean resulting in deep mixed layers (>50 m) in the Kimberley and Pilbara (Fig. 5a,e) and upwelling at Two Rocks (Fig. 5i) due to southerly (upwelling favourable) winds. Kimberley and Pilbara transects also show evidence of internal waves (Fig. 5a,e). This region has been documented to experience large internal waves that are generated by the semi-diurnal tide^34^. In autumn, Kimberley and Pilbara transects were very similar to that for summer, although there was no internal wave activity at Pilbara (Fig. 5f). At Two Rocks, DSWC was initiated (Fig. 5j) mainly due to reduction in wind speeds allowing for higher salinity water to exit the shelf^5^. During winter, DSWC was present along all three locations driven by surface cooling (Fig. 5c,g,k). In the Kimberley, tidal mixing allowed for vertical mixing of the water column in shallower waters and with increasing depth the diminishing influence of the tidal mixing allowed for the presence of DSWC (Fig. 5c). At all three locations denser water was present closest to the coast with vertically stratified water on the continental shelf. The cross-shelf density gradients at Kimberley, Pilbara and Two Rocks were: −0.512 × 10^−5^ kgm^−4^ (Fig. 5c), −1.423 × 10^−5^ kgm^−4^ (Fig. 5g), −1.768 × 10^−5^ kgm^−4^ (Fig. 5j), respectively. During spring there was no DSWC present at any of the locations with upwelling dominating (at Kimberley and Pilbara due to south-westerly winds and at Two Rocks due to southerly winds).

**Figure 6:**
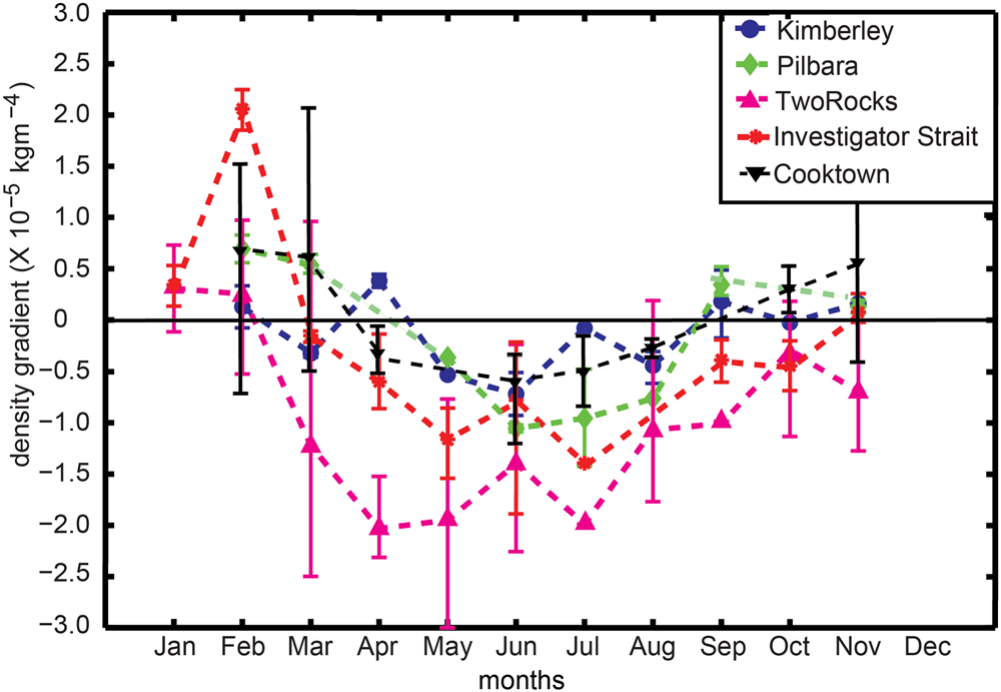
The monthly mean cross-shelf density gradient for Kimberley, Pilbara, Two Rocks, Investigator Strait and Cooktown. The mean density gradient was calculated using data for each month at each location over several years. The bars show the range of values calculated at each location reflecting the inter-annual variability.

The main driving force for DSWC formation was the cross-shelf density gradient ($\frac{\partial \rho }{\partial x}$; see Eqs. 2 and 6) and the ocean glider data were used to calculate the seasonal variability in $\frac{\partial \rho }{\partial x}$ (Fig. 6). In general, at all five locations, $\frac{\partial \rho }{\partial x}$ was negative during the cooler months showing the dominance of winter cooling in controlling $\,\frac{\partial \rho }{\partial x}$. During the austral summer months (January, February, no data were collected during December), $\frac{\partial \rho }{\partial x}$ was positive at all 5 locations and transited to negative values in March and remained negative to August, although at Two Rocks and Investigator Strait the negative $\frac{\partial \rho }{\partial x}$ persisted until November (Fig. 6). The timing of the minimum $\frac{\partial \rho }{\partial x}$ (i.e. maximum forcing) occurred in July at Kimberley and Pilbara, June in Investigator Strait and in May at Two Rocks. The maxima in May for Two Rocks reflect the combined influence of both salinity and temperature contributing to $\frac{\partial \rho }{\partial x}$^5^. The $\frac{\partial \rho }{\partial x}$was largest at Two Rocks when compared to other locations reaching a maximum of −3.0 × 10^−5^ kgm^−4^ whist at Investigator Strait, Pilbara and Kimberley the maximum values were: −1.878 × 10^−5^ kgm^−4^, −0.829 × 10^−5^ kgm^−4^ and −0.487 × 10^−5^ kgm^−4^, respectively (Table 1). The $\frac{\partial \rho }{\partial x}$along the east coast ranged between −0.982 × 10^−5^ kgm^−4^ (Capricorn Channel) and −0.366 × 10^−5^ kgm^−4^ (Port Stephens) (Table 1).

### Balance between stratification and mixing

**Figure 7:**
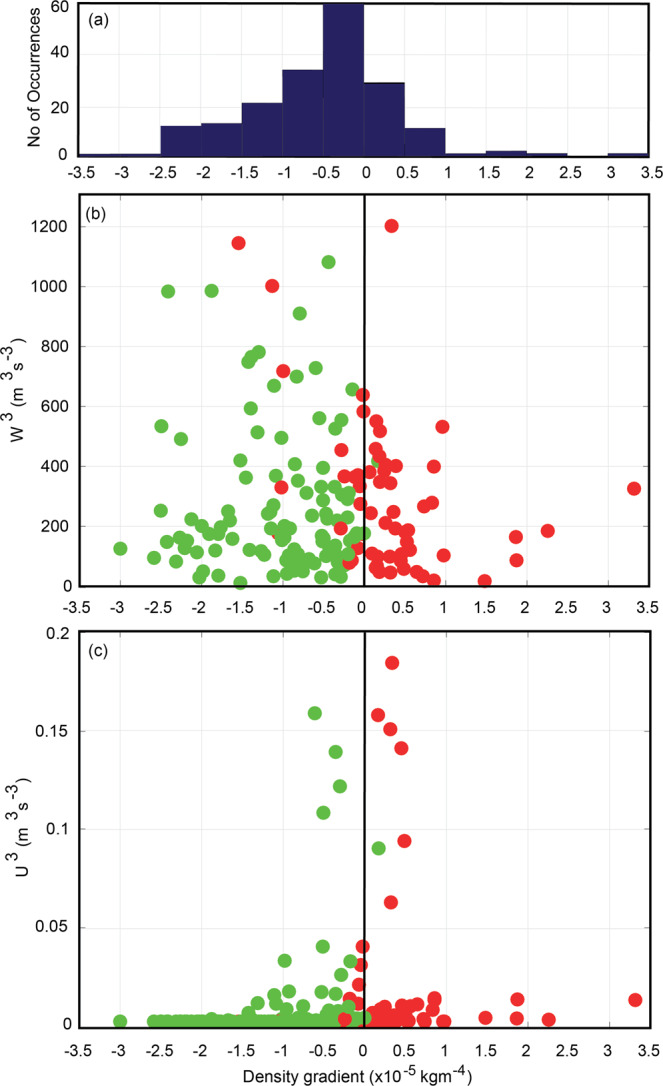
(**a**) Distribution of the number of occurrences for different cross-shelf density gradient; (**b**) scatter plot of cross-shelf density gradient and W^3^, a proxy for wind mixing; and, (**c**) scatter plot of cross-shelf density gradient and U^3^, a proxy for tidal mixing. Green (red) colour symbols indicate presence (absence) of DSWC.

Formation of DSWC is dependent on the ability of the buoyancy input (the stratifying force) through the cross-shelf density gradient $(\frac{\partial \rho }{\partial x})$ to overcome vertical mixing (destratifying force) provided by input of turbulent kinetic energy through wind and tidal action (Eqs. 2 and 6). Data from the 254 transects obtained under varying wind and tidal conditions were examined for the presence of DSWC and were related to local wind and tide conditions. The values of the individual terms in Eq. 6 are presented in Fig. 7. The observation sites in this study consisted of a gradual varying (small slope) continental shelves with mean depths of 40–50 m with exception of Investigator Strait which had a mean depth of 70 m. The cross-shelf density gradient, $\frac{\partial \rho }{\partial x}$was negative for majority (75%) of transects examined with ~ 50% of $\frac{\partial \rho }{\partial x}$ in the range 0 to −1.5 × 10^–5^ kgm^−4^ (Fig. 7a). Main variables in Eq. 6, $\frac{\partial \rho }{\partial x}$, W^3^ (proxy for wind mixing) and U^3^ (proxy for tidal mixing) were also evaluated to examine the relative magnitude of the terms and the presence of DSWC. The results indicated that the main parameter controlling the DSWC was $\frac{\partial \rho }{\partial x}$: when $\frac{\partial \rho }{\partial x}$ < 0, DSWC was present; whilst $\frac{\partial \rho }{\partial x}$ > 0 inhibited the presence of DSWC (Fig. 7b,c). The largest variation in tidal mixing energy was at Kimberley with a maximum tidal range of 10 m. Here, although $\frac{\partial \rho }{\partial x}$ was relatively small (>−1.0 × 10^–5^ kgm^−4^; Fig. 7c), DSWCs were present even when the maximum currents were up to 0.50 ms^−1^ (U^3^ = 0.15 m^3^s^−3^). At other locations, tidal mixing was negligible (Fig. 7c). Wind mixing included a range wind speeds (maxima > 15 ms^−1^) and, except for 5 points at Two Rocks, there was DSWC present whenever $\frac{\partial \rho }{\partial x}$ < 0. The 5 points where $\frac{\partial \rho }{\partial x}$ < 0 that did not indicate the presence of DSWC corresponded to winds associated with the passage of storm fronts that promoted downwelling^30^.

### DSWC as a conduit for chlorophyll, backscatter and dissolved oxygen

**Figure 8:**
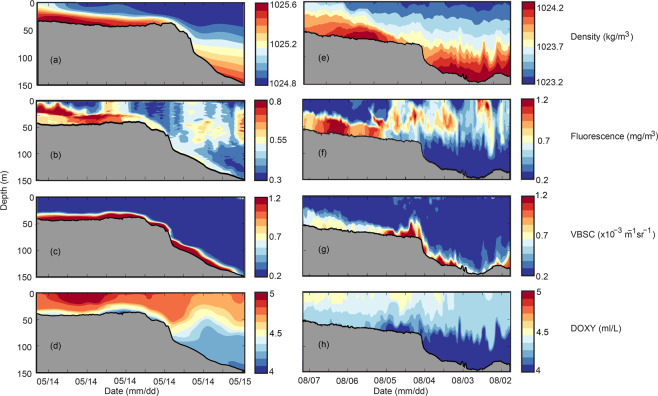
Water properties (density, fluorescence, backscatter and dissolved oxygen) measured by a Slocum ocean glider along: (**a-d**) Two Rocks transect during May 2016; and, (**e-h**) along the Pilbara transect during July 2012.

The Slocum ocean gliders contained a WETLabs BBFL2SLO 3 parameter optical sensor that measured chlorophyll fluorescence (a proxy for phytoplankton biomass), coloured dissolved organic matter (CDOM), and volume backscatter at 660 nm (a proxy for suspended material) and an Aanderaa dissolved oxygen (DO) Optode that collected data simultaneously with the temperature and salinity profiles^35,36^. Examples of these data streams from Two Rocks and Pilbara (Fig. 8) indicated that bottom layer associated with DSWC consisted of elevated fluorescence and backscatter values (Fig. 8b,c,f,g). The study region is strongly oligotrophic as there are no major terrestrial sources or upwelling for the supply of nutrients and therefore, water column nutrient concentrations are very low. There are no major river inputs resulting in very clear waters with photosynthesis occurring in depths up to 150 m^36^. In contrast to other regions there is no spring phytoplankton bloom; the seasonal chlorophyll concentrations show maxima during winter due to remobilisation of nutrients from sediments^37^. Fluorescence distribution in Pilbara indicated higher values in the bottom to water depths 70 m when there was an abrupt shift with higher values at the surface (Fig. 8f). This change was associated with a strong local horizontal density gradient in the bottom layer and elevated backscatter at the shelf edge (Fig. 8f), most likely due to breaking internal waves^38^. In terms of suspended material, at Two Rocks the backscatter signal extended to water depth of 150 m (Fig. 8c) subsequent to resuspension due to storm activity^36^. At Pilbara, DSWC contained elevated backscatter values across the shelf (Fig. 8g). A maxima in the backscatter was located at the shelf break due to resuspension by internal waves^38^. The DSWC was also associated with lower dissolved oxygen values (saturation levels ~ 75%) in the bottom layer. This was due to higher productivity of nutrient supply from the sediments^36,37^ and strong vertical stratification, due to the presence of DSWC inhibiting vertical mixing (Fig. 8d,h). At both locations the DSWC acted as a conduit to transfer chlorophyll and suspended matter across the continental shelf into deeper water (Fig. 8).

## Discussion

Terrestrial inputs from both natural and anthropogenic influences are discharged into the coastal margin and are transported along-shore and cross-shore by prevailing hydrodynamic conditions that are controlled by the local tidal, wind and buoyancy forcing^1,2^. In general, along-shore transport, with the land mass providing a barrier, is much stronger than the cross-shore, however, the weaker cross-shore transport is critical for the offshore transport of material from the coastal zone^2^.

Majority of coastal regions globally are dominated by freshwater inputs from rivers but regions, particularly those experiencing Mediterranean climates experience high evaporation during the summer months and cooling during the winter. Many of these regions are characterised by inverse estuaries and embayments where evaporation exceed freshwater input and the upper reaches contain denser water when compared to those at the seaward margin. As a result, differences in density, estuaries are characterized by longitudinal density gradients that may drive residual circulation with denser water flowing beneath less dense water under the influence of gravity^9^. In continental shelf regions similar processes operate and recent studies have documented this process, termed Dense Shelf Water Cascade (DSWC) from both field measurements^5,13,30,39–41^ and numerical model simulations^29,42,43^. These studies were limited to specific locations and did not examine the seasonal variability and its forcing mechanisms. In shallow continental shelf regions, the dynamical balance consists of buoyancy, Coriolis, and friction terms, and hence DSWC moves offshore at an angle to the coastline^23^. This paper used an extensive multi-year ocean glider data set obtained from 8 locations around the Australian continent under a range of incident tide and wind conditions that also enabled us to resolve seasonal variability.

In coastal and estuarine systems, vertical mixing by wind and tide acts to break down stratification and ultimately determines the vertical structure of the water column and thus the strength of the gravitational circulation^24,44^. Horizontal Richardson number (*Ri*_*x*_), also defined as the Simpson number (Eq. 1), is a measure of the balance between longitudinal density gradient (stratifying force) to vertical mixing (destratifying force)^24,26,27^. Evaluating Eq. 2 using data obtained from this study indicated that *Ri*_*x*_ was O(1) indicating dominance of stratification by horizontal density gradients over tidal and wind mixing leading to a stratified water column^26^.

Data presented in this study indicated that the presence of DSWC was controlled by the cross-shelf density gradient, $\frac{\partial \rho }{\partial x}$except under very strong wind speeds: when $\frac{\partial \rho }{\partial x}$ < 0, DSWC was present; whilst when $\frac{\partial \rho }{\partial x}$ > 0 there was no DSWC (Fig. 7b,c). The maximum $\frac{\partial \rho }{\partial x}$ recorded during this study was −3 × 10^–5^ kgm^−4^ (Fig. 6). A similar value was reported by Shearman and Brink^13^ for the north-west of Australia. In comparison, freshwater dominated systems such as the York River estuary^24^ (USA), $\frac{\partial \rho }{\partial x}$is a factor 10 larger at −30 × 10^–5^ kgm^−4^ and Liverpool Bay^45^ (UK) was −10 × 10^–5^ kgm^−4^. Coastal embayments that have inverse estuarine behaviour: Shark Bay, Spencer Gulf and the upper Gulf of California have values of −20, −5 and −6 × 10^–5^ kgm^−4^, respectively. In the mid-Atlantic Bight $\frac{\partial \rho }{\partial x}\,$ = −2 × 10^–5^ kgm^−4^ and was associated with winter cooling^43^. Thus although $\frac{\partial \rho }{\partial x}$ was the dominant factor that controlled the presence of DSWC the values recorded by the ocean glider data were comparable to those reported in open coastlines elsewhere. This leaves the possibility that as higher resolution data become available it is possible that many more regions with DSWC may be discovered.

Previous studies have shown that DSWC are persistent features that exist over extended periods at seasonal time scales. The cascades may be interrupted by local wind and tidal conditions that occur over periods of a few days but they reform after such events^30^. During these events, vertical mixing energy provided by tidal and/or wind action overcomes the buoyancy input through the cross-shelf density gradient. After the cessation of the event, buoyancy input dominates and DSWC is re-established^21^. The tidal and wind induced mixing terms (Eq. 6), represent scalar quantities and therefore do not include wind direction and/or changes in tidal range (e.g. spring-neap cycle). We now consider the influence of tidal range and wind direction on DSWC. Vertical mixing of the water column occurs through turbulent kinetic energy (TKE) generated by the action of tides and winds. Tidal action generates TKE at the seabed that is advected upwards from the seabed into the water column. In contrast, wind generates TKE at the air-sea interface that is advected downwards from the sea surface^6^. The main temporal variability in tidal mixing is due to the fortnightly spring-neap cycle with increased mixing energy during spring tides. If the tidal mixing energy is able to mix the whole water column then DSWC does not exist. However, at all of the locations examined here, particularly in the Kimberley region where the tidal range is 10 m and maximum bottom currents > 0.50 ms^-1^, DSWC were present irrespective of the tidal stage (i.e. within a tidal cycle or over the spring-neap cycle) indicating that buoyancy input was the dominant factor. The influence of the spring-neap cycle was to change the extent of DSWC above the seabed (smaller height during neap tides) indicating that the tidal mixing did not extend through the whole water column.

The influence of wind speed and direction also has an influence and can be summarised as follows^1,6,20,21,46^. in the presence of a negative cross-shelf density gradient (denser water near shore, lighter far from the coast), onshore (offshore) winds enhanced (decreased) the vertical stratification and decreased (increased) the cross-shelf density gradient. Mahjabin *et al*.^30^ characterised the influence of wind speed and direction on the occurrence of DSWC and concluded that in the presence of a negative cross-shelf density gradient (-d𝜌/dx), DSWC’s were always present under low wind conditions. When there was an onshore component of wind (downwelling favourable) the DSWC was enhanced through strengthening of the (-d𝜌/dx). With an offshore component of wind there was vertical mixing as denser water was advected over lower less dense water (‘straining’). The mixing events that interrupted the DSWC lasted only for a few days and after the cessation of the event the DSWC reformed.

Results of this study highlight a unique global feature that occurs in Australian continental shelves where continental scale forcing, at both spatial and temporal scales, dominate the meso-scale and/or local forcing. Typical spatial scales of wind forcing are < 500 km limited by the size of the atmospheric systems and the temporal scale is 5–10 days due to the passage of weather systems. There are large gradients in tidal forcing over spatial scales of ~ 1000 km. In Western Australia, over a 2000 km coastline tidal regime changes from semi-diurnal tides with a 10 m tidal range in the north to diurnal tides with 0.6 m tidal range in the south^5^. The temporal scale of the tides includes the fortnightly spring-neap cycle. Freshwater inputs are mainly point sources and their influence on the coastal system (ROFI) is generally limited to 100’s of km (<1000 km for the Mississippi). Across the Australian continent, the influence of evaporation and cooling cover a spatial distance > 10,000 km (Figs. 2 and S1, see Supplementary) and a time period of order months. Forcing through air-sea fluxes results in higher density water on the inner shelf particularly during the winter months providing the driving force for the formation of DSWC around the Australian coast. Here, the continental scale forcing through air-sea fluxes overcomes the local wind and tidal forcing.

Cross-shelf transport, to which DSWC is a strong contributor, has a major role in ecosystem functioning and bio-geochemical processes as a conduit for the transport of nearshore water and dissolved and suspended material off the continental shelves^2^. Instances of strong DSWC events transporting material over distances from the source region have been documented elsewhere^47^. In the Two Rocks region (Fig. 3) field measurements indicated that, within the DSWC, the mean cross-shelf currents (~ 0.10 ms^-1^) were comparable to the alongshore currents^30^. Often it is considered that along-shore currents are much stronger than the cross-shore currents^1,2^ and thus the higher cross-shore currents within the DSWC is unique. Higher concentrations of chlorophyll and suspended material together with offshore directed currents demonstrate that DSWC may have a major influence on the cross-shelf transport along coastlines extending over 10,000 km. This leads to the conclusion that during the winter months when the coastal regions in Australia are cooling due to heat loss, the inner shelf water exits along the seabed to deeper water, ‘draining’ the system.

## Conclusions

Dense shelf water is formed when the density of the inner shelf water is increased either by a decrease in temperature through cooling and/or an increase in salinity from evaporation. This cross-shelf density gradient drives a gravitational circulation with offshore transport of higher density water along the sea bed. This process is similar to that observed in ‘inverse’ estuarine systems and defined as Dense Shelf Water Cascades (DSWC). Analysis of an extensive multi-year data set obtained using ocean gliders confirmed that, under a range of wind and tidal conditions, DSWCs occurred regularly during autumn and winter months all around Australia’s continental shelves. It is concluded that the cross-shelf density gradient established across the continental shelf through air-sea fluxes (cooling and evaporation) is the primary forcing mechanism for the formation of the DSWC. Data indicated that even in the presence of relatively high wind- and tidally-induced vertical mixing, DSWCs were present due to the strength of the cross-shelf density gradient. Formation of DSWC around Australia is a unique occurrence globally where continental scale forcing through air-sea fluxes overcome the local wind and tidal forcing. The DSWC acts as a conduit for the transport of chlorophyll and suspended material across the continental shelf and is a critical process influencing cross-shelf transport that controls water quality on the inner continental shelf.

## Methods

Water column data were obtained from cross-shelf transects undertaken using Teledyne Webb Research Slocum electric gliders (http://www.webbresearch.com/) from 8 locations around Australia (Fig. 3). The ocean gliders are operated by Integrated Marine Observing System (IMOS) Ocean Glider facility hosted by The University of Western Australia^35^. All the ocean glider data are publicly available through the Australian Ocean Data Network (https://portal.aodn.org.au). Ocean gliders obtained data from the surface to 2 m above the seabed, travelling at a mean horizontal speed of 25 km per day^48^. The glider traverses a saw-tooth pattern using buoyancy control whilst moving forward to the target destination and navigates its way to a series of pre-programmed waypoints using GPS, internal dead reckoning and altimeter measurements. The gliders were equipped with a Sea-Bird Scientific SBE 41CP pumped CTD (conductivity–temperature–depth) sensor, a WETLabs BBFL2SLO 3 parameter optical sensor (which measured chlorophyll fluorescence, coloured dissolved organic matter, and backscatter at 660 nm), and an Aanderaa oxygen optode. All the sensors sampled at 4 Hz which yielded measurements 7 cm in the vertical. A single glider mission operated over a period of 25–30 days and traversed ~ 500–600 km and performed ~ 4000 vertical dives. During a single mission many repeat transects across the shelf were completed and depending on the width of the shelf each transect was completed over a period of 2–7 days. In water depths of 40–50 m, the glider takes ~ 20 mins to perform a single dive (i.e. from surface to bottom and return to surface) providing a vertical profile every 10 mins. The horizontal separation of the profiles was ~ 100 m and over a daily cycle ~ 140 vertical profiles were completed.

The focus of this paper is on the temperature and salinity (and density) data. Subsequent to the ocean glider recovery, all the data collected by the glider were subject to QA/QC procedures that include a series of automated and manual tests^49^. IMOS data streams are provided in NetCDF-4 format with data files containing meta-data and scientific data for each glider mission.

Ocean glider data collected between 2008 and 2019 from 8 different locations (Fig. 3) around the Australian continent were analysed and included 254 transects selected from 126 ocean glider deployments each lasting 20–25 days. This is equivalent to > 2700 days of continuous ocean profiles. The data set included a total of 275547 vertical profiles and over 84 million data scans in total. The analyses presented here is limited to the top 150 m of the water although the glider acquired data to a maximum depth of 200 m.

The horizontal density gradients were calculated by subtracting the depth-mean density at a point closest to the coast from the depth-mean density at a point at the seaward end of the transect and dividing by the distance between the two points. The resulting density gradient was defined to be negative when the nearshore water was denser than offshore.

The 8 locations around Australia (Fig. 3) were selected based on the availability of ocean glider data^35^. These locations have contrasting tidal (0.6 to 10 m tidal range) and wind (maximum wind speeds > 15 ms^-1^) forcing and include: (1) The Kimberley, north-west Australia: macro-tidal and moderate winds^13^; (2) The Pilbara, north-west Australia: macro-tidal^50^; (3) Two Rocks, Western Australia: wind dominated^21,41^ with low tidal range and diurnal tides^51^; (4) Investigator Strait, South Australia: tidally dominated^52,53^; (5) Port Stephens, New South Wales: macro tides^54^ and strong winds^55^; (6) Yamba, New South Wales: mostly wind driven and micro-tidal^56^; (7) Capricorn Channel, Queensland: moderate winds and macro tides^57^; and, (8) Cooktown, Queensland: moderate winds and macro tides^58,59^. A summary of the study locations and associated maximum cross-shore density gradients estimated from ocean glider data are shown in Table 1.

Tidal conditions were predicted using the TPXO7.2 global database (http://volkov.oce.orst.edu/tides/TPXO7.2.html) and wind data were obtained from the European Centre for Medium-Range Weather Forecast Interim Reanalysis (ECMWF ERA-I)^46^ (https://apps.ecmwf.int/datasets/data/interim-full-daily/levtype=sfc/). Terms in Eq. (3) were evaluated through estimates of local wind speed cubed (|W^3^ | ) and bottom current speed cubed (|U^3^ | ).

Although data from 8 locations were available; only 5 locations Kimberley, Pilbara, Two Rocks, Investigator Strait and Cooktown (Fig. 3) contained data throughout the whole year to define the seasonal variability. The mean horizontal density gradient for each month of the year was calculated using multiple transects acquired over several years. Two Rocks contained the highest repeating missions of ~ 44, and other locations: Kimberley, Pilbara, Investigator Strait and Cooktown consisted of 15, 12, 16 and 71 missions respectively. Error bars show minimum and maximum density gradients for each month at each of the 5 locations spread over different years (Fig. 6).

## Supplementary information

Supplementary Figures.

## Data Availability

All the data used in this paper are available from the data portal of the Integrated Marine Observing System (IMOS): https://portal.aodn.org.au/. Tidal data are predicted from TPXO7.2 global database and wind data are obtained from the European Centre for Medium-Range Weather Forecast Interim Reanalysis (ECMWF ERA-I).
