# Supplementary material for: Occurrence and seasonal variability of Dense Shelf Water Cascades along Australian continental shelves
Source: Sci Rep. 2020 Jun 16;10:9732. doi: 10.1038/s41598-020-66711-5 (PMC7298046; doi:10.1038/s41598-020-66711-5)
Supplement: Supplementary file 1 — Supplementary Figures. [file 41598_2020_66711_MOESM1_ESM.pdf]

Supplementary Material

Occurrence and seasonal variability of Dense Shelf Water Cascades along Australian continental shelves

Tanziha Mahjabin[1,*]**, Charitha Pattiaratchi**[1]**, and Yasha Hetzel**[1]

[1]Oceans Graduate School and The UWA Oceans Institute, The University of Western Australia, Crawley, WA 6009, Australia

[*]tanziha.mahjabin@research.uwa.edu.au

Contents of this file

Figure S1. And Figure S2.

Introduction

In this supporting information we provide details on

(1) Mean summer (January-February) and winter (May-June) net heat fluxes and freshwater budgets for the oceans around Australia obtained from Objectively Analyzed air-sea Heat Flux (OAFlux) (http://oaflux.whoi.edu/). OAFlux products are constructed from an optimal blending of satellite retrievals and atmospheric reanalyses and provides global time series of ocean latent and sensible heat fluxes, ocean evaporation, and flux-related surface meteorology from 1958 to 2018 (http://oaflux.whoi.edu/).

(2) Location of ocean glider transects.

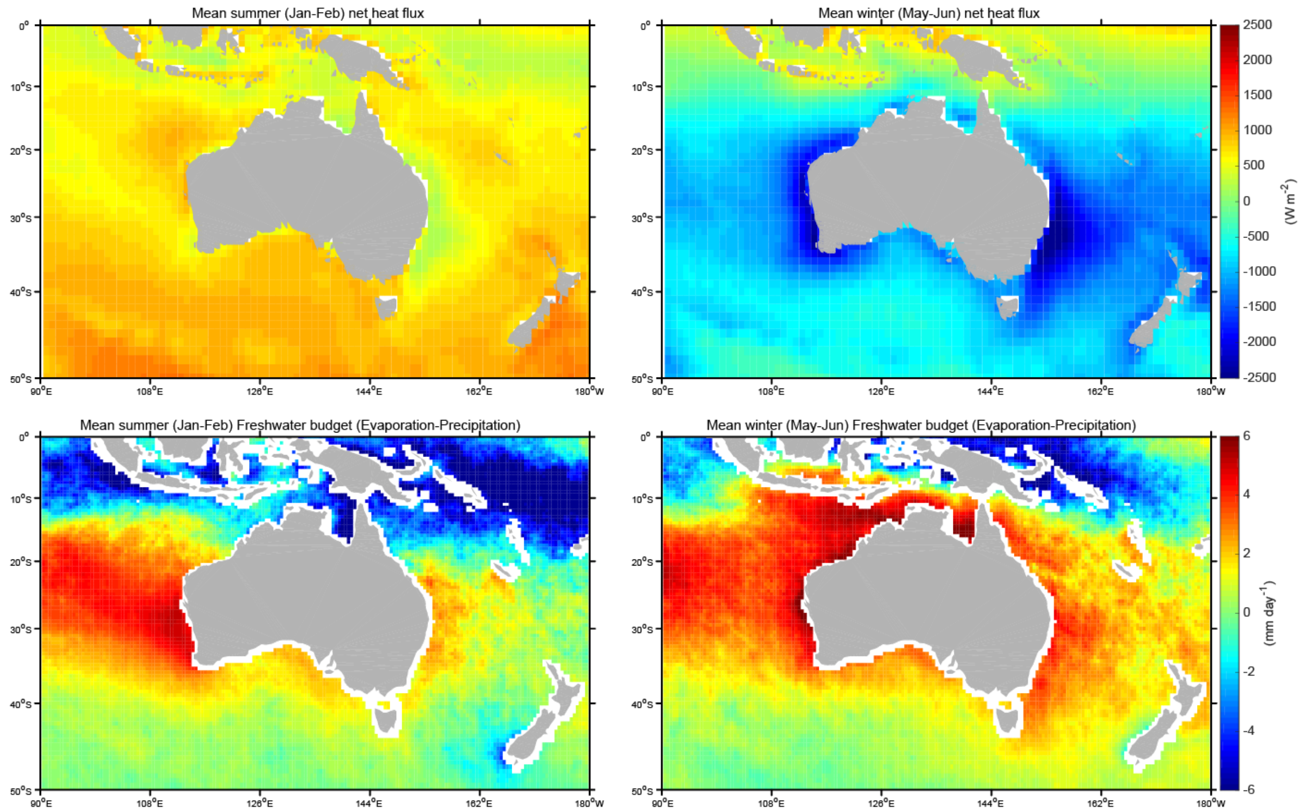

Figure S1. Mean summer (January-February) and winter (May-June) net heat fluxes and freshwater budgets for the oceans around Australia obtained from OAFlux products (http://oaflux.whoi.edu/).

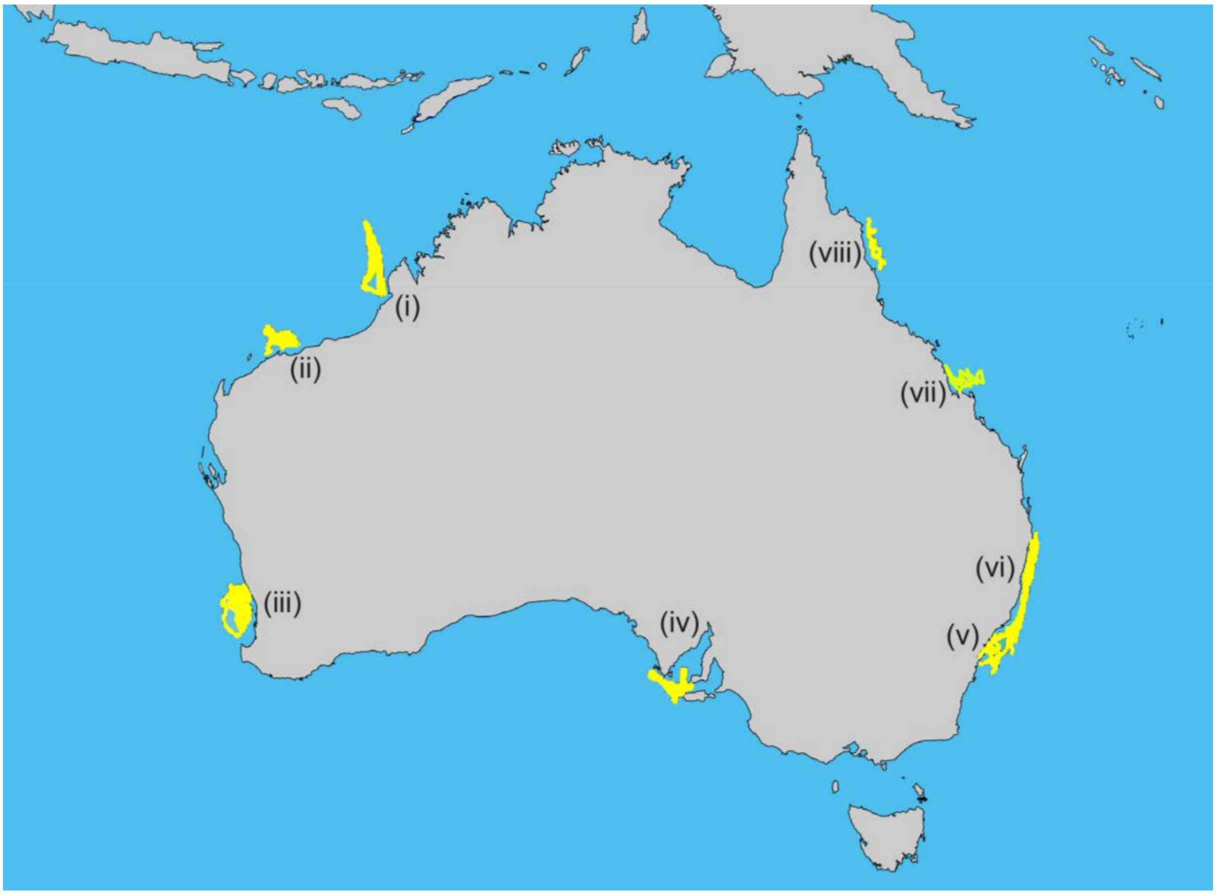

Figure S2. Locations of ocean glider transects around Australia used in the analysis. Designated locations are: (i) Kimberley, (ii) Pilbara, (iii) Two Rocks, (iv) Investigator Strait, (v) Port Stephens, (vi) Yamba, (vii) Capricorn Channel, (viii) Cooktown.
